# Inhibition of miR-214-3p Protects Endothelial Cells from ox-LDL-Induced Damage by Targeting GPX4

**DOI:** 10.1155/2021/9919729

**Published:** 2021-07-06

**Authors:** Min Xie, Panhao Huang, Tian Wu, Li Chen, Ren Guo

**Affiliations:** ^1^Department of Operating Room, The Third Xiangya Hospital, Central South University, Changsha, 410013 Hunan, China; ^2^Department of Pharmacy, The Third Xiangya Hospital, Central South University, Changsha, 410013 Hunan, China; ^3^Department of Infectious Diseases, The Third Xiangya Hospital, Central South University, Changsha, 410013 Hunan, China

## Abstract

It is generally believed that excessive production of reactive oxygen species (ROS) during cardiovascular diseases impairs endothelial function. In this study, we aimed to investigate whether miR-214-3p is involved in the endothelial dysfunction induced by oxidized low-density lipoprotein (ox-LDL). In cultured vascular endothelial cells (VECs), the effects of miR-214-3p on endothelial injury induced by 100 mg/L ox-LDL were evaluated by knockdown of miR-214-3p. Western blotting was used to determine the expression of glutathione peroxidase 4 (GPX4) and endothelial nitric oxide synthase (eNOS) in VECs under different conditions. A luciferase reporter assay was used to identify GPX4 as the target of miR-214-3p. Our data showed that 100 mg/L ox-LDL significantly decreased the expression of GPX4 and eNOS, which was associated with increases in ROS levels and impairments of VEC viability and migration. Knockdown of miR-214-3p could partially reduce the increase in ROS, restore the decreased expression of GPX4 and eNOS, and thus rescue the impaired endothelial function caused by ox-LDL. Our data demonstrated that ox-LDL could induce upregulation of miR-214-3p and result in suppression of GPX4 in VECs. Downregulation of miR-214-3p could protect VECs from ROS-induced endothelial dysfunction by reversing its inhibitory effect on GPX4 expression.

## 1. Introduction

At present, the incidence rates of chronic diseases such as coronary heart disease, hypertension, and diabetes are on the rise all over the world, seriously endangering human health [1]. Many studies have shown that these cardiovascular diseases are closely associated with endothelial dysfunction [2, 3]. Vascular endothelial cells (VECs) mainly locate in the inner surface of the vasculatures; they consist of a tightly connected layer to act as a barrier between the blood and vascular walls. The basic function of VECs is to act as a barrier between the blood and vascular walls. In addition, VECs can synthesize and release a variety of vasoactive mediators that can regulate vascular permeability, VEC-specific chemotaxis, vasoconstriction and vasodilatation, and platelet aggregation. It has been evident that many factors such as oxidized low-density lipoprotein (ox-LDL), angiotensin II (Ang II), and interleukin-6 (IL-6) can impair the barrier and secretion functions of VECs in different cardiovascular diseases, leading to an acceleration of disease progression [4]. For example, oxidative stress injury caused by ox-LDL is the most common mechanism of endothelial damage [5]. On the other hand, ox-LDL can also impair endothelial nitric oxide synthase (eNOS) function, leading to a reduction in the production of NO. VECs synthesize and release a series of vasoactive factors by sensing changes in the internal environment to maintain endothelial function and vascular homeostasis. The most widely studied of these protective factors secreted by VECs is NO, which is produced by eNOS through catalysis of L-arginine. NO exerts a strong effect on relaxing blood vessels and maintaining vascular physiological function. In cardiovascular disease studies, the expression of eNOS is often used as an important indicator to evaluate endothelial function [6].

Reactive oxygen species (ROS) are important components of free radicals necessary for cell biological behaviors, but excessive ROS production under pathological conditions often leads to endothelial dysfunction [7]. In the long process of evolution, the human body has developed an antioxidant system to protect cells from the damage caused by excessive ROS production. Glutathione peroxidase (GPX) is one of the three main nonenzymatic antioxidants in the human body [8]. Seven types of GPXs have been identified in humans, and GPX4 is the most researched type, showing the strongest antioxidative capacity [9]. The main oxidizing substrate of GPX4 is phospholipid hydroperoxide, which is present in biofilms. In addition, GPX4 can catalyze H_2_O_2_ and different lipid hydroperoxides, such as free fatty acid hydroperoxides, thymidine hydroperoxides, cholesterol, and cholesterol ester hydroperoxides. GPX4 has been shown to be associated with cardiovascular diseases, whereby overexpression of GPX4 in apolipoprotein E-deficient mice and in a myocardial ischemia/reperfusion mouse model inhibits the development of atherosclerosis and protects cardiac contractile function, respectively [10, 11]. Ectonucleotide pyrophosphatase 2 (ENPP2) is a lipid kinase that protects cardiomyocytes from erastin-induced ferroptosis by enhancing the expression of GPX4 [12].

MicroRNAs (miRNAs) are a class of small noncoding RNAs composed of 19-25 nucleotides. By recognizing and binding to the 3′-untranslated regions (UTRs) of target genes, miRNAs cause the degradation or block the translation of target genes. A large number of studies have confirmed that miRNAs are closely related to cell growth, differentiation, apoptosis, neural development, and oxidative stress. Abnormal expression of certain miRNAs in the circulation leads to a variety of diseases, such as atherosclerosis, tumors, diabetes, and Parkinson's disease [13–16]. Previous studies have documented that many miRNAs, such as miR-145 and miR-590, participate in the progression of vascular injury [17–19]. In our research, we found that miR-214-3p was also significantly upregulated in ox-LDL-induced VECs. Furthermore, by using bioinformatics methods, we found that GPX4 may be a direct target of miR-214-3p. Although miR-214-3p has been reported to play a role in colorectal cancer and the inflammatory pathogenesis of Parkinson's disease [20, 21], the mechanism by which miR-214-3p exerts its effects on VECs remains unclear. In this study, we aimed to investigate the relationship between miR-214-3p and GPX4 in an ox-LDL-induced VEC damage model.

## 2. Materials and Methods

### 2.1. Cell Culture

The human VEC line (human umbilical vein endothelial cell (HUVEC)) was purchased from the Cell Lab of Central South University and was cultured in Dulbecco's modified Eagle medium (DMEM, Gibco, USA) containing 10% fetal bovine serum (Gibco, USA) in a 5% CO_2_ incubator at 37°C. Subsequently, after 3 passages, the cells were placed in serum-free medium for 12 h prior to the experiment and then divided into different groups to receive different treatments. ox-LDL was purchased from Yiyuan Biotechnology (Guangzhou, China, No. YB-002). VECs were treated with 100 mg/L ox-LDL for 24 h to induce endothelial damage.

### 2.2. Transfection

First, the transfection complex was prepared as follows: 10× riboFECT buffer (RiboBio, China) was diluted 10 times, and then, 20 *μ*L miR-214-3p mimic or inhibitor was diluted in 120 *μ*L diluted riboFECT buffer to obtain the nucleic acid dilution. The sequences of miRNA-214-3p mimic and inhibitor are presented as follows: miR-214-3p mimics: 5′-ACAGCAGGCACAGACAGGCAG U-3′ (sense), 5′-UGCCUGUCUGUGCCUG CUGUUU-3′ (antisense); miR-214-3p inhibitor: 5′-ACUGCCUGUCUGUGCCUGCUGU-3′ (sense), 5′-CAGUACUUU UGUGUAG UACAA-3′ (antisense). Twelve microliters of transfection reagent was slowly added to the nucleic acid solution to obtain the transfection complex. To overexpress or knock down the expression of miR-214-3p, the transfection complexes with miR-214-3p mimic or inhibitor were added to the cell culture medium, and then, the cells were incubated in a 5% CO_2_ incubator at 37°C for 24 h. 50 nmol/L miR-214-3p mimic and 100 nmol/L miR-214-3p inhibitor were used to treating VECs.

### 2.3. Real-Time PCR

Total RNA from VECs was extracted for reverse transcription and real-time quantitative PCR. Real-time PCR was performed according to the manufacturer's instructions using the ABI 7500 real-time PCR system with SYBR Green reagents. The primers for miR-214-3p are listed as follows: miR-214-3p-F: ACAGCAGGCACAGACAGGCAG; miR-214-3p-R: GTGCAGGGTCCGAGGTAT TC. The CT values of each sample were normalized to the U6 values as an internal reference, and the expression levels of miR-214-3p were calculated by the 2^-*ΔΔ*CT^ method. All amplification reactions were performed in triplicate.

### 2.4. Western Blotting

Protein was extracted from VECs using RIPA buffer and phenylmethanesulfonyl fluoride (PMSF). Then, 40 *μ*g of total protein was separated by 10% SDS-PAGE, and the protein bands were then transferred to a polyvinylidene difluoride membrane (Millipore, USA). After blocking with 5% nonfat milk in TBST, the membrane was incubated overnight with rabbit anti-human GPX4 (polyclonal antibody, Sigma, USA, Catalog Number: SAB2108670) and eNOS antibodies (polyclonal antibody, Sigma, USA, Catalog Number: SAB4502013) overnight. Immunoreactive bands were visualized by electrochemiluminescence (Bio-Rad, USA) after incubation with a horseradish peroxidase-conjugated anti-mouse secondary antibody. The ratio of the protein of interest to GAPDH was used for statistical analysis.

### 2.5. Dual-Luciferase Reporter Assay

Two human GPX4 3′-UTR sequences containing predicted miR-214-3p target sites and the mutant 3′-UTR of GPX4 were synthesized and inserted into the pGL3 control vector (Promega, USA). For the reporter assay, VECs were seeded into 24-well plates and cotransfected with 200 ng pGL3-GPX4-3′-UTR with 100 nmol/L miR-214-3p mimics or a negative control. Cells cotransfected with 200 ng pGL3-mut-GPX4-3′-UTR plasmid with 100 nmol/L miR-214-3p mimics or negative control were used as controls. X-tremeGENE HP DNA Transfection Reagent (Roche, Switzerland) was used for transfection of pGL3 report plasmid into VECs. After incubation for 24 h, luciferase activities were measured using a dual-luciferase reporter assay kit (Promega, USA) according to the manufacturer's instructions.

### 2.6. Cell Viability Test

VECs were cultured to logarithmic growth phase and seeded on 96-well plates at a cell density of 10^4^ and then cultured in a constant temperature incubator at 37°C and 5% CO_2_ for 24 h. A 50 *μ*L 1× MTT solution was added to each well, and the plate was placed in a cell incubator at 37°C for 4 h. The cell supernatants were discarded, 150 *μ*L DMSO was added to each well, and the plate was vibrated for 10 min on an oscillator. The absorbance value (OD) of each well was detected by a Bio-Rad 680 microplate analyzer at a wavelength of 570 nm, and the average OD value of three replicate wells was taken.

### 2.7. ROS Detection

An ROS kit from Beyotime Biotechnology was used to detect the ROS level in cells following the manufacturer's instructions. First, 10^6^ cells were seeded on a plate and incubated in a 5% CO_2_ incubator at 37°C for 24 h. Then, DCFH-DA was diluted 1 : 1000 with serum-free medium to a final concentration of 10 *μ*mol/L. An appropriate volume of diluted DCFH-DA was added to the cell culture medium. Then, the cell culture plate was placed into a 37°C incubator for 20 min. The cells were washed with serum-free cell culture solution 3 times to remove the DCFH-DA that did not enter the cells. Finally, flow cytometry (Beckman, USA) was used to detect the intensity of intracellular green fluorescence. FlowJo software was used to quantize the fluorescence intensity to evaluate the ROS level.

### 2.8. Statistical Analysis

SPSS software (version 16.0) was used for the statistical analysis. The data are expressed as the mean ± SD. The differences among the groups were compared using one-way ANOVA. *P* < 0.05 was considered to be statistically significant.

## 3. Results

### 3.1. ox-LDL Induced an Increase in miR-214-3p Expression and a Decrease in GPX4 Expression

**Figure 1:**
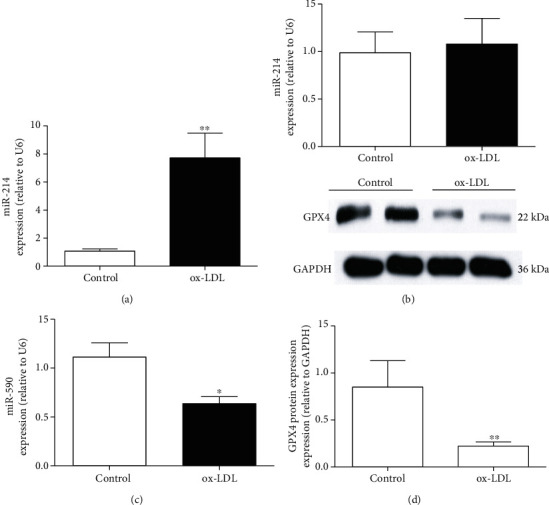
The expression of miR-214-3p and GPX4 in ox-LDL-stimulated VECs. The expressions of miR-214 (a), miR-145 (b), and miR-590 (c) after treatment with 100 mg/L ox-LDL in VECs. All values are expressed as the mean ± SD, *n* = 3. ^∗^*P* < 0.05 and ^∗∗^*P* < 0.01, compared with the control group.

To determine the changes in miR-214-3p after 100 mg/L ox-LDL stimulation and its influence on VECs, we first measured the expression of miR-214-3p in VECs after ox-LDL treatment. Real-time PCR revealed that ox-LDL caused an approximately 8-fold increase in miR-214-3p expression compared with the control treatment (Figure 1(a)). We also observed that ox-LDL treatment did not cause significant changes in miR-145 but significantly reduced the expression level of miR-590 (Figures 1(b) and 1(c)). Since the endothelial protective effect of miR-590 has been elucidated in the paper by Wu et al., our study mainly focused on miR-214-3p [19]. Interestingly, we also observed a significant decrease in GPX4 protein expression upon ox-LDL treatment of VECs (Figure 1(d)). Together, these results suggest that both miR-214-3p and GPX4 are involved in ox-LDL-induced damage to VECs.

### 3.2. GPX4 Is a Target of miR-214-3p

**Figure 2:**
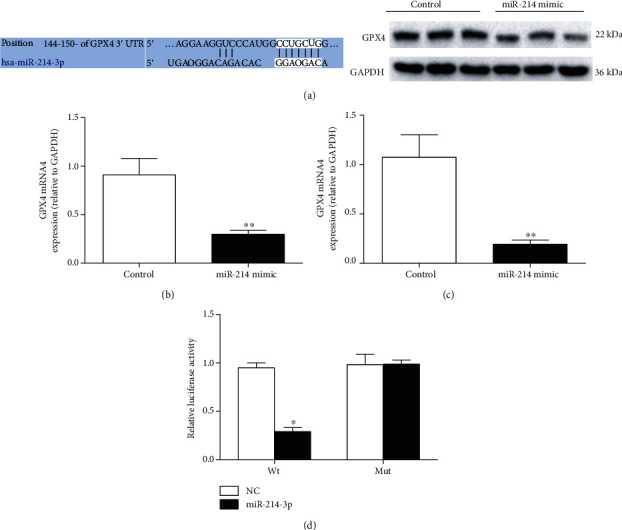
GPX4 is a target of miR-214-3p. (a) Predicted binding sites of miR-214-3p to GPX4; (b) real-time PCR measurement after miR-214-3p mimic treatment; (c) representative Western blot and statistical analyses of GPX4 in VECs treated with 100 nmol/L miR-214-3p mimic; (d) luciferase activity assay after miR-214-3p mimic treatment. All values are expressed as the mean ± SD, *n* = 3. ^∗^*P* < 0.05 and ^∗∗^*P* < 0.01, compared with the control group.

We used the online software TargetScan 7.2 to explore the possible association of miR-214-3p with GPX4. As shown in Figure 2(a), the 3′-UTR of GPX4 contained possible binding sites for miR-214-3p. Then, 100 nmol/L miR-214-3p mimic was used to overexpress miR-214-3p in VECs. As shown in Figures 2(b) and 2(c), overexpression of miR-214-3p by the miR-214-3p mimic significantly decreased GPX4 expression at both the mRNA and protein levels. Furthermore, a luciferase activity assay showed that the miR-214-3p mimic significantly inhibited the luciferase activity of the wild-type but not the mutant 3′-UTR of the GPX4 gene in VECs (Figure 2(d)). These data suggest that GPX4 is a direct target of miR-214-3p.

### 3.3. Suppression of miR-214-3p Rescues VEC Viability upon ox-LDL Treatment

**Figure 3:**
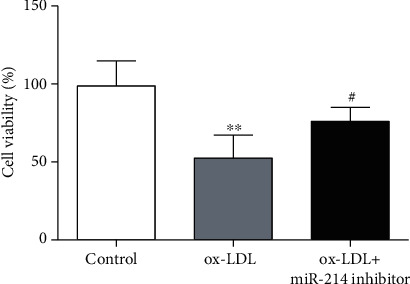
Suppression of miR-214-3p rescues VEC viability upon ox-LDL treatment. All values are expressed as the mean ± SD, *n* = 3. ^∗^*P* < 0.05, compared with the control group; ^#^*P* < 0.05, compared with the ox-LDL treatment group.

Because the process of ox-LDL-induced endothelial damage was accompanied by the upregulation of miR-214-3p expression, we next determined the influence of miR-214-3p on the ox-LDL-induced inhibition of endothelial activity by reducing the level of miR-214-3p. The MTT test demonstrated that 100 mg/L ox-LDL caused a significant decrease in VEC viability, whereas pretreatment of VECs with 200 nmol/L miR-214-3p inhibitor partially rescued the impaired VEC growth in comparison with the control treatment (Figure 3).

### 3.4. Inhibition of miR-214-3p Reverses the Decreased Expression of GPX4 and eNOS upon ox-LDL Treatment

**Figure 4:**
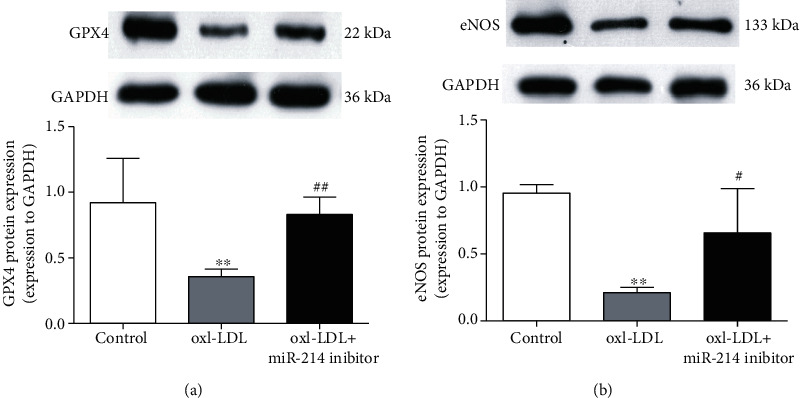
Inhibition of miR-214-3p reverses the decreased expression of GPX4 and eNOS upon ox-LDL treatment. (a) Representative Western blot and statistical analyses of VECs treated either with 100 mg/L ox-LDL or 100 mg/L ox-LDL+miR-214-3p inhibitor for GPX4; (b) representative Western blot and statistical analyses of eNOS in VECs treated either with 100 mg/L ox-LDL or 100 mg/L ox-LDL+miR-214-3p inhibitor. All values are expressed as the mean ± SD, *n* = 3. ^∗∗^*P* < 0.01, compared with the control group; ^#^*P* < 0.05 and ^##^*P* < 0.01, compared with the ox-LDL treatment group.

GPX4 expression and activity are crucial for inhibiting the level of vascular oxidative stress. eNOS is the primary enzyme that produces NO and is also very important for the maintenance of normal vasodilation function. Therefore, in this study, we also investigated the effect of miR-214-3p on GPX4 and eNOS after ox-LDL stimulation. Treatment of VECs with ox-LDL for 24 h decreased the expression of GPX4 and eNOS, whereas miR-214-3p inhibitor-mediated depletion of miR-214-3p partially restored the expression of GPX4 and eNOS compared with that in the control group (Figures 4(a) and 4(b)).

### 3.5. Inhibition of miR-214-3p Restores the Impaired VEC Migration Induced by ox-LDL

**Figure 5:**
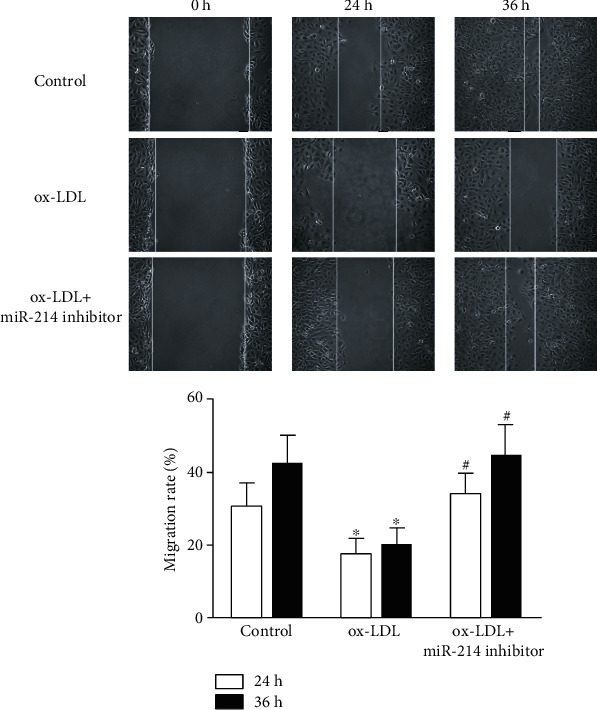
Depletion of miR-214-3p rescues ox-LDL-induced VEC migration impairments. Representative images of VECs treated either with 100 mg/L ox-LDL or 100 mg/L ox-LDL+miR-214-3p inhibitor for migration. All values are expressed as the mean ± SD, n = 3. ^∗^*P* < 0.05, compared with the control group; ^#^*P* < 0.05, compared with the ox-LDL treatment group. Scale bar: 100 *μ*m.

In addition to cell viability, we also observed VEC migration under different treatments. As shown in Figure 5, ox-LDL treatment of VECs significantly impaired the migration ability of VECs within 24 h, as indicated by the longer distance between the isolated cells. However, compared with the control treatment, miR-214-3p inhibitor-mediated depletion of miR-214-3p partially restored the migration ability of VECs.

### 3.6. miR-214-3p Affects ROS Levels in VECs upon ox-LDL Treatment

**Figure 6:**
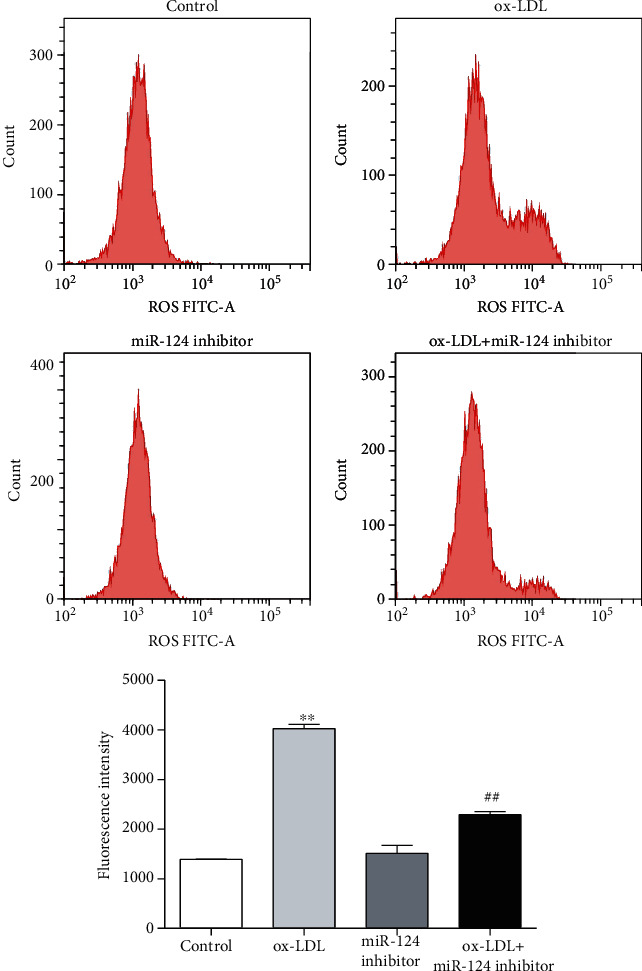
miR-214-3p upregulates the level of ROS in VECs upon ox-LDL treatment. Representative images of ROS levels in VECs treated with either 100 mg/L ox-LDL or 100 mg/L ox-LDL+miR-214-3p inhibitor. All values are expressed as the mean ± SD, *n* = 3. ^∗∗^*P* < 0.01, compared with the control group; ^##^*P* < 0.01, compared with the ox-LDL treatment group. Scale bar: 100 *μ*m.

High levels of ROS are often associated with endothelial dysfunction, so we also explored whether knockdown of miR-214-3p could affect ROS levels in VECs stimulated by ox-LDL. Our data indicated that ox-LDL induced a dramatic increase in ROS levels in VECs, and pretreatment of VECs with the miR-214-3p inhibitor could partially lower the increased ROS levels compared with those in the control group (Figure 6).

## 4. Discussion

In this study, we used ox-LDL to establish an endothelial damage model. It is generally believed that ox-LDL-induced endothelial damage is the initial event of atherosclerosis and is involved in the entire process of atherosclerosis, including the formation and rupture of atherosclerotic plaques. Our data suggest that 100 *μ*g/mL ox-LDL significantly increased the ROS level and impaired the viability and migration of cultured VECs. To determine the potential mechanism by which ox-LDL triggers the increase in ROS levels, GPX4 expression was detected because of its important role in the antioxidant system. As expected, treatment of VECs with ox-LDL decreased GPX4 expression, which was accompanied by VEC dysfunction and increased ROS levels. Although many studies have revealed that the normal expression and activity of GPX4 are critical for maintaining oxidative homeostasis in different cell types [22–24], one study also showed that the mRNA levels of GPX1, GPX3, and GPX4 were significantly upregulated in patients with acute coronary syndrome (ACS) [25]. It is known that ox-LDL, TNF-*α*, IL-6, and other atherosclerosis stimuli remain at a high level in ACS patients for a long time, which may lead to the adaptive upregulation of the expression and activity of antioxidant enzymes in the affected cells. However, this adaptive regulation of GPX4 is not sufficient to effectively remove the excessive levels of ROS.

After determining the decreased expression of GPX4 in VECs induced by ox-LDL, we next attempted to find the factor responsible for the decreased GPX4 expression upon stimulation with ox-LDL. Previous studies have demonstrated that many miRNAs exhibit oxidative stress-related expression changes in atherosclerosis and hypertension [18]. Therefore, in this study, we detected several miRNAs with previously reported vasoprotective effects, including miR-145, miR-590, and miR-214-3p. Our data showed that treatment of VECs with ox-LDL triggered significant decreases in miR-590 and miR-214-3p expression levels. Previously, Wu and his colleagues reported that miR-590 could protect VECs from Ang II-induced damage by inhibiting the expression of CD40 [19]. Therefore, we mainly focused on the association of miR-214-3p with GPX4 during ox-LDL-induced endothelial dysfunction. Interestingly, by using the online software TargetScanHuman 7.1, we found that GPX4 may be a target gene of miR-214-3p. To determine the regulation of GPX4 by miR-214-3p, we overexpressed miR-214-3p by using a miR-214-3p mimic to investigate its effect on GPX4 expression. Our results indicated that miR-214-3p overexpression significantly decreased GPX4 expression in normal conditions but not in ox-LDL-induced conditions, which is likely because the strong suppression of GPX4 exerted by ox-LDL masks the effect of miR-214-3p overexpression. Furthermore, in our luciferase reporter assay, we found that miR-214-3p inhibited the luciferase activity of the wild-type but not the mutant 3′-UTR of the GPX4 gene in VECs, which provided direct evidence that GPX4 is a target gene of miR-214-3p.

Having demonstrated that GPX4 is a direct target gene of miR-214-3p, we sought to determine whether inhibiting miR-214-3p expression could be a feasible way to rescue the impaired endothelial function upon ox-LDL stimulation. Excessive ROS production in cardiovascular diseases often causes endothelial dysfunction, which is manifested as decreased viability, migration, and NO production of VECs [26–28]. Here, we observed that inhibition of miR-214-3p expression by a miR-214-3p inhibitor rescued the observed endothelial dysfunction, which showed that VEC viability and migration ability were improved under ox-LDL stimulation. NO produced by eNOS is the most important endothelium-derived relaxing factor to maintain normal vasodilation. Furthermore, ROS derived from eNOS uncoupling also show severe toxic effects on VECs during different disease states. Therefore, we investigated eNOS expression upon stimulation with ox-LDL and miR-214-3p inhibitor. Consistent with previously published data, 100 mg/L ox-LDL led to a significant decrease in eNOS expression, which was partially reversed by pretreatment of VECs with the miR-214-3p inhibitor.

Our study reported that the expression of the vascular enriched miRNA miR-214-3p is stimulated by ox-LDL. Downregulating miR-214-3p expression under ox-LDL conditions can protect VECs from ROS-induced endothelial dysfunction, likely by reversing the miRNA-mediated inhibition of GPX4 expression. These results, together with previous findings showing the effects of miRNAs on the vasculature, suggest that miR-214-3p and other miRNAs represent novel therapeutic targets for cardiovascular diseases.

However, the current study also has some limitations. For example, GPX4 is an antioxidant enzyme whose expression and activity both affect its ability to clear ROS. However, this study mainly focused on the expression change of GPX4 after ox-LDL administration. In addition, it has been recently reported that GPX4 is involved in the process of ferroptosis, a newly identified metabolic stress-induced programmed cell death that is different from apoptosis. Further research is also needed to investigate the possible association of miR-214-3p with ferroptosis in cardiovascular disease.

## Data Availability

The datasets used and/or analyzed during the current study are available from the corresponding author on reasonable request.
